# Engineering of a Potent Recombinant Lectin-Toxin Fusion Protein to Eliminate Human Pluripotent Stem Cells

**DOI:** 10.3390/molecules22071151

**Published:** 2017-07-10

**Authors:** Hiroaki Tateno, Fumi Minoshima, Sayoko Saito

**Affiliations:** Biotechnology Research Institute for Drug Discovery (BRD), National Institute of Advanced Industrial Science and Technology (AIST), Tsukuba Central 2, 1-1-1 Umezono, Tsukuba, Ibaraki 305-8568, Japan; fumi-minoshima@aist.go.jp (F.M.); sayoko.saitou@aist.go.jp (S.S.)

**Keywords:** lectin, pluripotent stem cells, tumorigenicity, regenerative medicine

## Abstract

The use of human pluripotent stem cells (hPSCs) such as human embryonic stem cells (hESCs) and human induced pluripotent stem cells (hiPSCs) in regenerative medicine is hindered by their tumorigenic potential. Previously, we developed a recombinant lectin-toxin fusion protein of the hPSC-specific lectin rBC2LCN, which has a 23 kDa catalytic domain (domain III) of *Pseudomonas aeruginosa* exotoxin A (rBC2LCN-PE23). This fusion protein could selectively eliminate hPSCs following its addition to the cell culture medium. Here we conjugated rBC2LCN lectin with a 38 kDa domain of exotoxin A containing domains Ib and II in addition to domain III (PE38). The developed rBC2LCN-PE38 fusion protein could eliminate 50% of 201B7 hPSCs at a concentration of 0.003 μg/mL (24 h incubation), representing an approximately 556-fold higher activity than rBC2LCN-PE23. Little or no effect on human fibroblasts, human mesenchymal stem cells, and hiPSC-derived hepatocytes was observed at concentrations lower than 1 μg/mL. Finally, we demonstrate that rBC2LCN-PE38 selectively eliminates hiPSCs from a mixed culture of hiPSCs and hiPSC-derived hepatocytes. Since rBC2LCN-PE38 can be prepared from soluble fractions of *E. coli* culture at a yield of 9 mg/L, rBC2LCN-PE38 represents a practical reagent to remove human pluripotent stem cells residing in cultured cells destined for transplantation.

## 1. Introduction

Human pluripotent stem cells (hPSCs) such as human embryonic stem cells (hESCs) [1] and human induced pluripotent stem cells (hiPSCs) [2] have immense potential as cell sources for regenerative medicine due to their self-renewal and pluripotency. However, major hurdles related to their tumorigenicity need to be overcome before they can be used for regenerative medicine purposes [3,4,5]. Given that as few as 100 transplanted hPSCs were demonstrated to form teratoma in a mouse model [6,7], the complete elimination of residual cells is therefore essential before hPSC-derived therapeutic cells can be used in clinical applications.

Previously, we found by lectin microarray that a lectin designated rBC2LCN (recombinant N-terminal domain of BC2L-C lectin derived from *Burkholderia cenocepacia*) binds selectively to hPSCs, but not to non-hPSCs [8]. From a practical perspective, fluorescently-labeled rBC2LCN could be used as a reagent for the identification of hPSCs by flow cytometry analysis and microscopic observation [9]. One of the biggest advantages of rBC2LCN is that the lectin can be used to strongly stain live hPSCs following its addition to the cell culture medium [9]. This staining is specific to hPSCs, and rapidly diminishes upon their differentiation. rBC2LCN binds to the Fucα1-2Galβ1-3 motif that is highly expressed on hPSCs [8,10]. Interestingly, rBC2LCN was found to be endocytosed inside hPSCs upon binding to these cells. Taking advantage of this finding, we previously generated a recombinant lectin-toxin fusion protein by fusing rBC2LCN with the 23 kDa catalytic domain of *Pseudomonas aeruginosa* exotoxin A (PE), termed rBC2LCN-PE23, for the targeted elimination of hPSCs [11]. hiPSCs and hESCs were completely eliminated when treated for 24 h with 10 μg/mL of rBC2LCN-PE23.

To create more-potent reagents to eliminate hPSCs, here rBC2LCN is fused with a 38 kDa domain of PE containing domains Ib and II in addition to domain III (PE38) [12]. The developed rBC2LCN-PE38 exhibited a robust cytotoxic effect on hPSCs compared to rBC2LCN-PE23. A concentration of rBC2LCN-PE38 as low as 0.003 μg/mL in the culture medium is sufficient for the 50% elimination of 201B7 hiPSCs, corresponding to a 556-fold higher toxicity against 201B7 hiPSCs than rBC2LCN-PE23. rBC2LCN-PE38 could thus be a cost-effective reagent to eliminate hPSCs present in hPSC-based cell therapy products.

## 2. Results

### 2.1. Production of rBC2LCN-PE38

**Figure 1:**
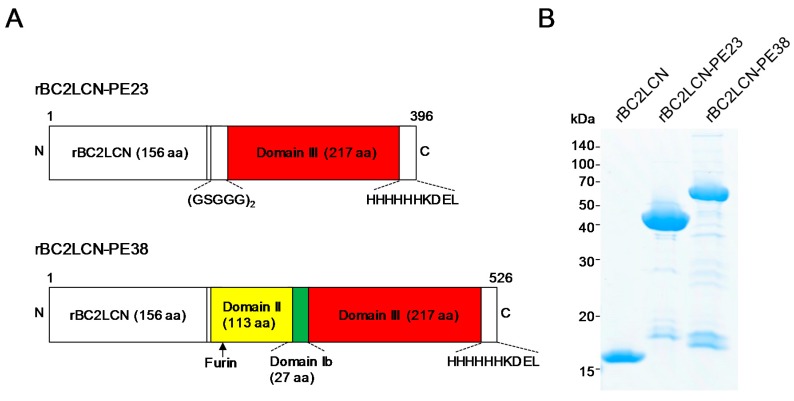
Production of rBC2LCN-PE38. (**A**) Domain structure of rBC2LCN-PE38 compared to rBC2LCN-PE23; (**B**) SDS-PAGE of purified rBC2LCN, rBC2LCN-PE23, and rBC2LCN-PE38. Four micrograms of purified rBC2LCN, rBC2LCN-PE23, or rBC2LCN-PE38 in the presence of 2-mercaptoethanol (2ME) were run on a 5–20% acrylamide gel and stained with Coomassie G-250.

Previously, we developed rBC2LCN-PE23, in which rBC2LCN was fused with a 23 kDa domain of PE, termed PE23, containing only domain III [11]. To increase the cytotoxicity of hPSCs, rBC2LCN (156 aa) was fused with a longer, 38 kDa domain (PE38) containing domains II (113 aa) and Ib (27 aa) in addition to domain III (217 aa) (Figure 1A) [12]. The generated rBC2LCN-PE38 (526 aa) was expressed in *E. coli* and purified by affinity chromatography, with a yield obtained of 9 mg/L of bacterial culture. rBC2LCN-PE38 gave a major band at a higher molecular weight of 54 kDa relative to rBC2LCN (16 kDa) and rBC2LCN-PE23 (42 kDa) on SDS-PAGE under reducing conditions (Figure 1B).

### 2.2. Glycan-Binding Properties of rBC2LCN-PE38

**Figure 2:**
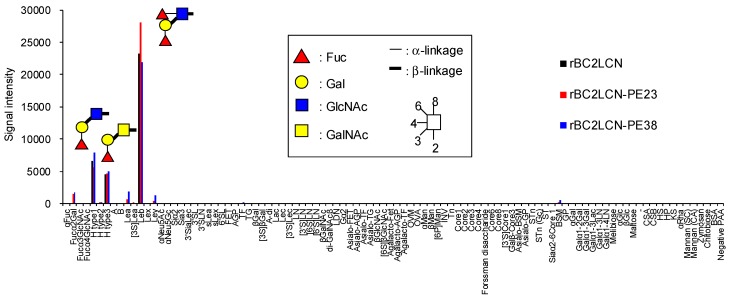
Glycan binding specificity of rBC2LCN-PE38. Cy3-labeled rBC2LCN, rBC2LCN-PE23, and rBC2LCN-PE38 were reacted with glycoconjugate microarray at 0.125 μg/mL overnight and scanned with an evanescent-field fluorescence scanner. Averages of the signals of triplicate spots are shown.

We analyzed by glycoconjugate microarray the glycan-binding properties of rBC2LCN-PE38 compared to wild-type rBC2LCN and rBC2LCN-PE23 [13]. rBC2LCN-PE38 exhibited a similar glycan-binding specificity to both rBC2LCN and rBC2LCN-PE23, and bound to Fucα1-2Galβ1-3 motif-containing polyacrylamide (PAA) probes such as Fucα1-2Galβ1-3GlcNAc-PAA (H type1), Fucα1-2Galβ1-3GalNAc-PAA (H type3), and Fucα1-2Galβ1-3(Fucα1-4)GlcNAc-PAA (Le^b^) (Figure 2 and Table S1). The binding affinity of rBC2LCN-PE38 was also evaluated by quantitative analysis with frontal affinity chromatography [14]. The association constant (*K*_a_) of rBC2LCN-PE38 for H type1-*p* nitrophenol (*p*NP) and H type3-*p*NP was 1.1 × 10^5^ and 3.3 × 10^4^ M^−1^, respectively, which were similar to that of wild-type rBC2LCN (H type1-*p*NP: 1.0 × 10^5^ M^−1^, H type3-*p*NP: 3.1 × 10^4^ M^−1^) [11]. Based on these results, we concluded that rBC2LCN-PE38 exhibits similar glycan-binding properties to wild-type rBC2LCN.

### 2.3. Quantitative Effect of rBC2LCN-PE38 on hiPSCs and Non-hPSCs

**Figure 3:**
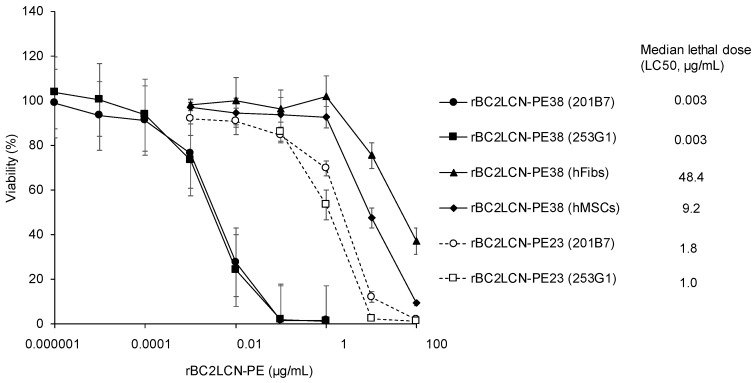
Quantitative effect of rBC2LCN-PE38 on the cell viability of human induced pluripotent stem cells (hiPSCs). 201B7 hiPSCs, 253G1 hiPSCs, human dermal fibroblasts (hFibs), and human adipose-derived mesenchymal stem cells (hMSCs) were cultured with varying concentrations of rBC2LCN-PE38 or rBC2LCN-PE23. After 24 h, the medium was replaced with fresh medium supplemented with 20 μL of Cell Counting Kit (CCK)-8 solution, and the absorbance at 450 nm was measured after a further 4 h incubation. Viability (%) was calculated from the absorbance at 450 nm of treated cells relative to untreated cells. Data are shown as mean ± SD (for triplicate measurements). Median lethal dose (LC50) was calculated by GraphPad Prism7 software.

We examined the cytotoxic effect of rBC2LCN-PE38 on hiPSCs and compared this to rBC2LCN-PE23. 201B7 hiPSCs were cultured with varying concentrations of rBC2LCN-PE23 or -PE38 (0~100 μg/mL) for 24 h, and the viability of the cells was quantitated by measuring the metabolic activity of living cells. As shown in Figure 3, median lethal dose (LC50) of rBC2LCN-PE23 to 201B7 hiPSCs was 1.8 μg/mL, whereas that of rBC2LCN-PE38 was 0.003 μg/mL. This indicates a 556-fold higher cytotoxicity of rBC2LCN-PE38 for hiPSCs than rBC2LCN-PE23. LC50 of rBC2LCN-PE38 for 253G1 hiPSCs was also similar (0.003 μg/mL) (Figure 3).

We also examined the effect of rBC2LCN-PE38 on the viability of non-hPSCs such as human dermal fibroblasts (hFibs) and human adipose-derived mesenchymal stem cells (hMSCs). LC50 of rBC2LCN-PE38 to hFibs and hMSCs was 48.4 and 9.2 μg/mL, respectively, which correspond to 14,606- and 2784-fold less active than to 201B7 hiPSCs (Figure 3).

### 2.4. Effect of rBC2LCN-PE38 on hiPSCs and Non-hPSCs Analyzed by Microscopy

**Figure 4:**
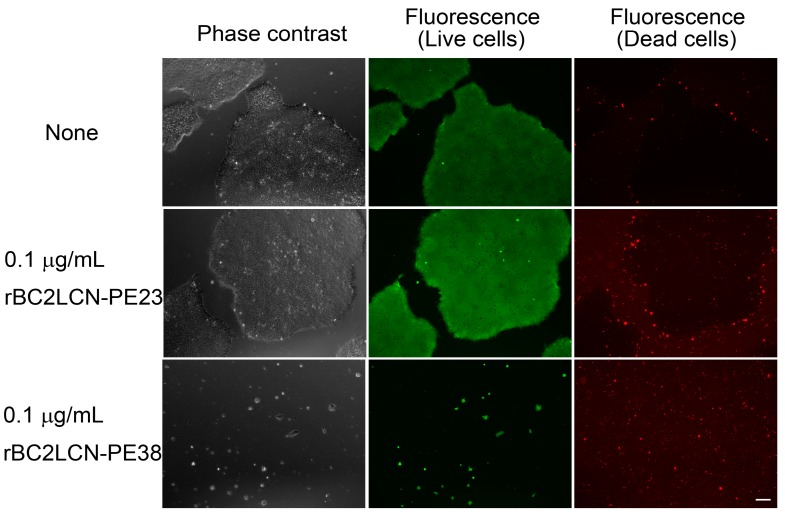
Effect of rBC2LCN-PE38 on the viability of hiPSCs. 201B7 hiPSCs were cultured with or without 0.1 μg/mL of rBC2LCN-PE23 or rBC2LCN-PE38. After 24 h, the cells were stained and observed with the aid of fluorescence microscopy. The live cell component showed cytoplasmic green fluorescence, whereas dying or dead cells exhibited nuclear red fluorescence. Scale bar: 100 μm.

**Figure 5:**
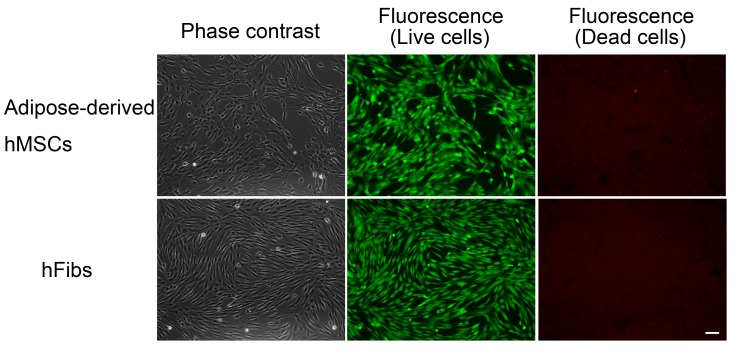
Effect of rBC2LCN-PE38 on the viability of differentiated cells. Human adipose-derived mesenchymal stem cells (hMSCs) and human dermal fibroblasts (hFibs) were cultured with 1 μg/mL of rBC2LCN-PE38. After 24 h, cells were stained and observed by fluorescence microscopy. Live cells exhibited cytoplasmic green fluorescence, whereas dying or dead cells showed nuclear red fluorescence. Scale bar: 100 μm.

The effect of rBC2LCN-PE23 and rBC2LCN-PE38 on hiPSCs was also analyzed by cell staining and microscopy observations (Figure 4). 201B7 hiPSCs were cultured for 24 h with 0.1 μg/mL of rBC2LCN-PE23 or rBC2LCN-PE38 and cells were then stained using a LIVE/DEAD Cell Imaging Kit. Live cells are stained with cytoplasmic green fluorescence, and dying or dead cells are stained with nuclear red fluorescence. When 201B7 hiPSCs were incubated with 0.1 μg/mL of rBC2LCN-PE23, the green fluorescence-positive live cells proliferated normally, and only a small number of red-stained dead cells could be identified, in agreement with the results obtained by metabolic measurement (Figure 3). In contrast, the green fluorescence-positive 201B7 hiPSCs disappeared when they were cultured in the presence of 0.1 μg/mL rBC2LCN-PE38 for 24 h. Most of the dying or dead cells had detached from the cell culture flasks and were floating in the medium. In contrast, both hFibs and hMSCs proliferated normally and exhibited green fluorescence, but not red fluorescence, even after 24 h treatment with 1 μg/mL rBC2LCN-PE38 (Figure 5). These results demonstrate that rBC2LCN-PE38 has a negligible cytotoxic effect on non-hPSCs such as hMSCs and hFibs.

### 2.5. Selective Elimination of hiPSCs Resident among hiPSC-Derived Hepatocytes

**Figure 6:**
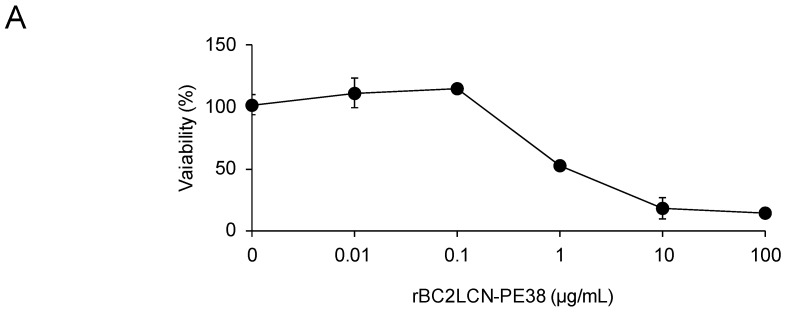

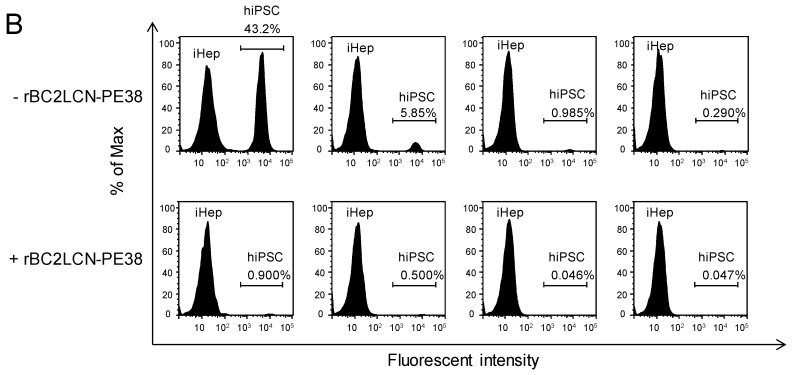
Selective killing by rBC2LCN-PE38 of hiPSCs resident among hiPSC-derived hepatic cells. (**A**) Effect of rBC2LCN on the viability of hiPSC-derived hepatocytes. 201B7 hiPSCs were cultured with different concentrations of rBC2LCN-PE38. After 24 h, the medium was replaced with fresh medium supplemented with 20 μL of CCK-8 solution, and the absorbance at 450 nm was measured after a further 4 h incubation. Viability (%) was calculated from the absorbance at 450 nm of treated cells relative to untreated cells. Data are shown as mean ± SD (for triplicate measurements); (**B**) 201B7 hiPSCs were cultured and fluorescently labeled with 20 μM of CellTracker™ Green CMFDA. After washing to remove residual fluorescence, hiPSC-derived hepatocytes (iHep) were mixed in different percentages and co-cultured in either the presence (**lower panels**) or absence (**upper panels**) of 0.1 μg/mL of rBC2LCN-PE38. After 24 h, flow cytometry data were acquired.

Finally, we attempted to eliminate hPSCs present among hiPSC-derived hepatocytes. First, we analyzed the effect of rBC2LCN-PE38 on hiPSC-derived hepatocytes, which were cultured with varying concentrations of rBC2LCN-PE38 (0–100 μg/mL) for 24 h and their viability then quantitated by measuring the metabolic activity of living cells. As shown in Figure 6A, no effect on the viability of hiPSC-derived hepatocytes was observed at concentrations of rBC2LCN-PE38 up to 0.1 μg/mL, but viability decreased at concentrations higher than 1 μg/mL. We therefore decided to use a concentration of 0.1 μg/mL to eliminate hiPSCs present among hiPSC-derived hepatocytes. 201B7 hiPSCs cultured on 12-well plates were labeled with CellTracker™ Green CMFDA, and after removing residual fluorescence, fluorescently-labeled 201B7 hiPSCs were co-cultured at different percentages with hiPSC-derived hepatocytes in either the presence or absence of 0.1 μg/mL rBC2LCN-PE38. After 24 h, cells were recovered and analyzed by flow cytometry (Figure 6B). In the absence of rBC2LCN-PE38, fluorescently-labeled 201B7 hiPSCs and unlabeled hiPSC-derived hepatocytes were detected in different ratios (43.2–0.29% of 201B7 hiPSCs) (Figure 6B, top). In the presence of rBC2LCN-PE38, however, fluorescently-labeled 201B7 hiPSCs had been almost completely removed (Figure 6B, bottom). These results demonstrate that rBC2LCN-PE38 can selectively eliminate hiPSCs, even in a mixed cell population with different percentages of differentiated hiPSC-derived hepatocytes.

## 3. Discussion

We have shown here that a fusion protein consisting of rBC2LCN with a 38 kDa PE domain—termed rBC2LCN-PE38—could be successfully produced in a fully active form from soluble fractions of *E. coli* culture medium. rBC2LCN-PE38 retained a glycan-binding activity similar to that of wild-type rBC2LCN and rBC2LCN-PE23, even though the molecular size of PE38 (38 kDa) is much larger than that of rBC2LCN lectin (16 kDa). In addition, the yield of rBC2LCN-PE38 (9 mg per liter of culture medium) was similar to that of rBC2LCN-PE23 (10 mg/L). Notably, the generated rBC2LCN-PE38 showed an approximately 556-fold higher cytotoxic activity to 201B7 hiPSCs than the previously developed rBC2LCN-PE23 [11]. PE is composed of 613 amino acids containing three domains: domain Ia with receptor binding activity, domain II with translocation activity, and domains Ib and III with ADP-ribosyltransferase activity. PE23 contains only domain III, whereas PE38 contains domain II as well as domains Ib and III. Therefore, the higher cytotoxic activity of rBC2LCN-PE38 depends largely on the presence of domains II and Ib. Although the functions of these two domains are not clearly understood, they were reported to be required for cell killing activity [12]. PE38 is the most commonly employed cytotoxic fragment of PE. Several immunotoxins incorporating PE38 have already reached the clinical trial stage for treating B-cell malignancies, lung cancer, and hematologic malignancies [12].

rBC2LCN-PE38 was produced in *E. coli*. Recombinant proteins produced in *E. coli* might contain residues of *E. coli*, such as lipopolysaccharide. Although wild-type rBC2LCN prepared in *E. coli* gave no visible toxicity to hiPSCs [9], we should pay attention in terms of toxicity when we use rBC2LCN-PE38 for cell culture or regenerative medicine.

Hepatocyte transplantation is one of the most attractive approaches for the treatment of patients with liver failure. However, donor sources of human hepatocytes are limited, and thus novel sources of hepatocytes are highly necessary. From this perspective, hepatocytes derived from hiPSCs are attractive cell sources for hepatocyte transplantation, since hiPSCs can be proliferated in a perpetual manner. One of the major concerns for the use of hiPSC-derived hepatocytes, however, is that tumorigenic hiPSCs residing in the hiPSC-derived hepatocyte population could form tumors in transplanted patients. We showed here that 0.1 μg/mL of rBC2LCN-PE38 could selectively remove hiPSCs from a mixed cell population of hiPSCs and hiPSC-derived hepatocytes. Since rBC2LCN-PE38 shows a 556-fold higher cytotoxicity to hPSCs compared to rBC2LCN-PE23, and their production yields are similar, rBC2LCN-PE38 could be expected to be a cost-effective reagent to remove hPSCs from hPSC-derived cell therapy products.

## 4. Materials and Methods

### 4.1. Construction, Expression, and Purification of rBC2LCN-PE38

A 38 kDa domain of PE (PE38) containing domain Ib (27 aa), domains II (113 aa), and domain III (217 aa) was inserted via *NheI* and *XhoI* into an expression vector of pET27b at the C-terminus of an N-terminal domain of BC2L-C identified from *Burkholderia cenocepacia* (rBC2LCN) (1–156 aa) (Figure 1) (Weldon and Pastan, 2011). A C-terminal 6xHis tag (PPHHHHHH) was also added for protein purification in case the expressed recombinant lectin could not be purified by sugar-immobilized column from the soluble fractions of *E. coli*, and endoplasmic reticulum (ER) retention signal sequence (KDEL) for efficient intracellular trafficking to the ER. The total number of amino acids was 526, and the calculated average molecular weight was 55,666 Da. The generated rBC2LCN-toxin fusion protein was designated “rBC2LCN-PE38”.

The expression plasmid of rBC2LCN-PE38 was transformed into *E. coli* BL21 CodonPlus (DE3)-RIL. The transformed *E. coli* was cultured in L-Broth medium containing 10 μg/mL kanamycin at 37 °C until the optical density (OD) at 600 nm reached 0.4. rBC2LCN-PE38 expression was induced by the addition of 1 mM IPTG at 20 °C for 24 h. The following procedures were performed at 4 °C. The *E. coli* cells were harvested by centrifugation at 4450× *g* for 30 min and lysed by sonication in phosphate buffered saline containing EDTA and Triton X-100 (PBSET: 6 mM Na_2_HPO_4_·12H_2_O, 1.4 mM KH_2_PO_4_, 140 mM NaCl pH 7.0, 1 mM EDTA, 0.1% Triton X-100) containing a protease inhibitor cocktail (Nacalai tesque). After centrifugation at 24,910× *g* for 30 min, supernatants were applied onto l-fucose-Sepharose and the bound rBC2LCN-PE38 was eluted with 0.2 M L-fucose in PBSE (6 mM Na_2_HPO_4_·12H_2_O, 1.4 mM KH_2_PO_4_, 140 mM NaCl pH 7.0, 1 mM EDTA). The purified rBC2LCN-PE38 was dialyzed against PBS. The protein concentration was measured by BCA protein assay (Thermo Scientific), and the purity was analyzed by electrophoresis using 17% XV pantera MP Gel (DRC).

### 4.2. SDS-PAGE

Four micrograms of purified lectins were incubated at 95 °C for 5 min in the presence of 2-mercaptoethanol and run on a 5–20% polyacrylamide gel (DRC). The gel was then stained with Bio-Safe Coomassie G-250 Stain (Bio-Rad, Hercules, CA, USA).

### 4.3. Frontal Affinity Chromatography

The principle and protocol for frontal affinity chromatography have been described previously [14,15]. Briefly, rBC2LCN-PE38 was immobilized onto NHS-activated Sepharose 4FF (GE) and packed into a miniature column (inner diameter, 2 mm; length, 10 mm, bed volume, 31.4 µL; Shimadzu, Kyoto, Japan) and connected to a high-performance liquid chromatograph (Shimadzu, Kyoto, Japan). Varying concentrations of H type1-*p*NP and H type3-*p*NP were injected into the column, and the elution front was determined relative to that of negative control LacNAc (*N*-acetyl lactosamine)-*p*NP, referred to V–V_0_. Finally, B_t_ (nmol) and *K*_d_ (μM) values were obtained from Woolf-Hofstee plots.

### 4.4. Cell Culture

201B7 and 253G1 hiPSCs were cultured in mTeSR1 (StemCell Technologies, Vancouver, BC, Canada) on cell culture plates coated with Matrigel (BD Biosciences) [2,16]. The human ES cell line, H1, was cultured in mTeSR1 according to the WiCell Feeder Independent Pluripotent Stem Cell Protocols provided by the WiCell Research Institute (www.wicell.org). 201B7 and H1 hESCs were differentiated by culturing them in mTeSR1 containing 10 µM of retinoic acid for 10 days. hFibs and hADSCs were cultured in MesenPRO RS medium. Cells were counted with a hemocytometer, a Vi-CELL Cell Viability Analyzer (Beckman coulter, Tokyo, Japan), or a TC20 Automated Cell Counter (Bio-Rad, Hercules, CA, USA). hiPSCs and hESCs used in this study were confirmed to have the ability to form teratoma and to be positive for both anti-SSEA4 and rBC2LCN [17].

### 4.5. Cell Viability Assay

The cytotoxicity of rBC2LCN-PE38 was analyzed using a LIVE/DEAD Cell Imaging Kit (Molecular Probes). Cells were cultured in mTeSR1 on 12-well plates coated with Matrigel in the presence or absence of rBC2LCN-PE38 (0.1 and 1 μg/mL). After 24 h, they were stained with Live Green (A) and Dead Red (B) reagents according to the manufacturer’s instructions and observed under an Axio Vert.A1 (Carl Zeiss, Oberkochen, Germany) or BZ-9000 fluorescence microscope.

The cytotoxicity of rBC2LCN-PE38 or –PE23 was also analyzed using a Cell Counting Kit-8 (Dojindo). Cells were cultured in 500 μL mTeSR1 on 24-well plates coated with Matrigel in the presence of different concentrations of rBC2LCN-PE38. After 24 h, the culture medium was replaced with 200 μL of fresh medium and 20 μL of CCK-8 solution, and after a further 4 h incubation in a CO_2_ incubator, the absorbance was measured at 450 nm. Median lethal dose (LC50) was calculated by GraphPad Prism7 software (GraphPad Software, Inc., La Jolla, CA, USA).

### 4.6. Flow Cytometry

201B7 hiPSCs were cultured in mTeSR1 on 12-well plates coated with Matrigel. Adherent cells were fluorescently labeled with 20 μM CellTracker™ Green CMFDA (Life Technologies, Carlsbad, CA, USA) and incubated at 37 °C for 45 min. After replacing the medium, cells were further cultured at 37 °C for 30 min. Unlabeled hiPSC-derived hepatocytes were seeded and co-cultured in either the presence or absence of 10 μg/mL of rBC2LCN-PE38. After 24 h, cells were recovered and centrifuged at 5000 rpm for 1 min. After removing the supernatant, the cells were suspended in 1 mL of PBS containing 1% bovine serum albumin. Finally, flow cytometry data were acquired on a CytoFLEX (BECKMAN COULTER) and analyzed using FlowJo software (FLOWJO, Ashland, OR, USA).

## 5. Conclusions

In conclusion, we have generated a lectin-toxin fusion protein by fusing an hPSC-specific lectin with a 38 kDa PE, termed rBC2LCN-PE38, which showed 556-fold higher cytotoxic activity to 201B7 hiPSCs compared to the previously developed rBC2LCN-PE23. Since rBC2LCN-PE38 can be produced in an *E. coli* expression system at a yield of 9 mg/L, this lectin-toxin fusion protein could serve as a practical reagent to remove hPSCs from cultured cells prior to the use of differentiated cells in regenerative medicine applications.

## Supplementary Materials

The supplementary materials are available online.
