# Supplementary material for: Engineering of a Potent Recombinant Lectin-Toxin Fusion Protein to Eliminate Human Pluripotent Stem Cells
Source: Molecules. 2017 Jul 10;22(7):1151. doi: 10.3390/molecules22071151 (PMC6152053; doi:10.3390/molecules22071151)
Supplement: Supplementary file 1 [file molecules-22-01151-s001.pdf]

Table S1, related to Figure 1. Glycans used for glycoconjugate microarray.

Number	Trivial name	Presentation	Glycans	Co.	Cat#
1	αFuc	PAA	Fucα1-PAA	Glycotech	01-007
2	Fucα2Gal	PAA	Fucα1-2Galβ1-PAA	Glycotech	01-019
3	Fucα3GlcNAc	PAA	Fucα1-3GlcNAcβ1-PAA	Glycotech	01-024
4	Fucα4GlcNAc	PAA	Fucα1-4GlcNAcβ1-PAA	Glycotech	01-025
5	H type1	PAA	Fucα1-2Galβ1-3GlcNAcβ1-PAA	Glycotech	01-037
6	H type2	PAA	Fucα1-2Galβ1-4GlcNAcβ1-PAA	Glycotech	08-034
7	H type3	PAA	Fucα1-2Galβ1-3GalNAcα1-PAA	Glycotech	08-060
8	A	PAA	GalNAcα1-3(Fucα1-2)Galβ1-4GlcNAcβ1-PAA	Glycotech	08-091
9	B	PAA	Galα1-3(Fucα1-2)Galβ1-4GlcNAcβ1-PAA	Glycotech	08-092
10	Le^a	PAA	Galβ1-3(Fucα1-4)GlcNAcβ1-PAA	Glycotech	01-035
11	[3S]Le^a	PAA	(3OSO$_3$)Galβ1-3(Fucα1-4)GlcNAcβ1-PAA	Glycotech	01-040
12	Le^b	PAA	Fucα1-2Galβ1-3(Fucα1-4)GlcNAcβ1-PAA	Glycotech	08-042
13	Le^x	PAA	Galβ1-4(Fucα1-3)GlcNAcβ1-PAA	Glycotech	01-036
14	Le^y	PAA	Fucα1-2Galβ1-4(Fucα1-3)GlcNAcβ1-PAA	Glycotech	08-043
15	αNeu5Ac	PAA	Neu5Acα2-PAA	Glycotech	01-012
16	αNeu5Gc	PAA	Neu5Gcα2-PAA	Glycotech	01-051
17	Sia2	PAA	Neu5Acα2-8Neu5Acα2-PAA	Glycotech	08-064
18	Sia3	PAA	Neu5Acα2-8Neu5Acα2-8Neu5Acα2-PAA	Glycotech	01-081
19	3'SiaLe^c	PAA	Neu5Acα2-3Galβ1-3GlcNAcβ1-PAA	Glycotech	01-078
20	3'SL	PAA	Neu5Acα2-3Galβ1-4Glcβ1-PAA	Glycotech	01-038
21	3'SLN	PAA	Neu5Acα2-3Galβ1-4GlcNAcβ1-PAA	Glycotech	01-077
22	sLe^a	PAA	Neu5Acα2-3Galβ1-3(Fucα1-4)GlcNAcβ1-PAA	Glycotech	08-044
23	sLe^x	PAA	Neu5Acα2-3Galβ1-4(Fucα1-3)GlcNAcβ1-PAA	Glycotech	01-045
24	6'SL	PAA	Neu5Acα2-6Galβ1-4Glcβ1-PAA	Glycotech	01-039
25	FET	Glycoprotein	Fetuin (Complex-type N-glycans and O-glycans)	Sigma	F3004
26	AGP	Glycoprotein	α1-acid glycoprotein (Complex-type N-glycans)	Sigma	G9885
27	TF	Glycoprotein	Transferrin (Complex-type N-glycans)	Sigma	T3309
28	TG	Glycoprotein	Porcine thyroglobulin (Complex and high-mannnose-type	Sigma	T1126
29	βGal	PAA	Galβ1-PAA	Glycotech	01-004
30	[3S]βGal	PAA	(3OSO$_3$)Galβ1-PAA	Glycotech	01-015
31	A-di	PAA	GalNAcα1-3Galβ1-PAA	Glycotech	01-017
32	Lac	PAA	Galβ1-4Glcβ1-PAA	Glycotech	01-021
33	Le^c	PAA	Galβ1-3GlcNAcβ1-PAA	Glycotech	01-020
34	[3'S]Le^c	PAA	(3OSO$_3$)Galβ1-3GlcNAcβ1-PAA	Glycotech	01-062
35	LN	PAA	Galβ1-4GlcNAcβ1-PAA	Glycotech	01-022
36	[3'S]LN	PAA	(3OSO$_3$)Galβ1-4GlcNAcβ1-PAA	Glycotech	01-061
37	[6S]LN	PAA	Galβ1-4(6OSO$_3$)GlcNAcβ1-PAA	Glycotech	01-066
38	[6'S]LN	PAA	(6OSO$_3$)Galβ1-4GlcNAcβ1-PAA	Glycotech	01-068
39	βGalNAc	PAA	GalNAcβ1-PAA	Glycotech	01-011
40	di-GalNAcβ	PAA	GalNAcβ1-3GalNAcβ1-PAA	Glycotech	01-070
41	LDN	PAA	GalNAcβ1-4GlcNAcβ1-PAA	Glycotech	01-057
42	GA2	PAA	GalNAcβ1-4Galβ1-4Glcβ1-PAA	Glycotech	08-074
43	Asialo-FET	Glycoprotein	Asialo fetuin (Desialylated complex-type N- and O-glycans)	Sigma	F3004 (Acid-
44	Asialo-AGP	Glycoprotein	Asialo α1-acid glycoprotein (Desialylated complex-type N-	Sigma	G9885 (Acid-
45	Asialo-TF	Glycoprotein	Asialo transferrin (Desialylated complex-type N-glycans)	Sigma	T3309 (Acid-
46	Asialo-TG	Glycoprotein	Asialo porcine thyroglobulin (Desialylated complex-type	Sigma	T1126 (Acid-
47	βGlcNAc	PAA	GlcNAcβ1-PAA	Glycotech	01-009
48	[6S]βGlcNAc	PAA	(6OSO$_3$)GlcNAcβ1-PAA	Glycotech	01-016
49	Agalacto-Fet	Glycoprotein	Agalacto fetuin (Agalactosylated complex-type N- and O-	Sigma	F3004
50	Agalacto-AGP	Glycoprotein	Agalacto α1-acid glycoprotein (Agalactosylated complex-	Sigma	G9885
51	Agalacto-TF	Glycoprotein	Agalacto transferrin (Agalactosylated complex-type N-	Sigma	T3309
52	OVM	Glycoprotein	Ovomucoid (Complex-type N-glycans)	Sigma	T2011
53	OVA	Glycoprotein	Ovoalbumin (Hybrid-type N-glycans)	Sigma	A2512
54	αMan	PAA	Manα1-PAA	Glycotech	01-005
55	βMan	PAA	Manβ1-PAA	Glycotech	01-050
56	[6P]Man	PAA	(6OPO$_4$)Manα1-PAA	Glycotech	01-006
57	INV	Glycoprotein	Yeast invertase (High mannose-type N-glycans)	Sigma	I4504
58	Tn	PAA	GalNAcα1-PAA	Glycotech	01-010
59	Core1	PAA	Galβ1-3GalNAcα1-PAA	Glycotech	08-023
60	Core2	PAA	Galβ1-3(GlcNAcβ1-6)GalNAcα1-PAA	Glycotech	01-083
61	Core3	PAA	GlcNAcβ1-3GalNAcα1-PAA	Glycotech	01-071
62	Core4	PAA	GlcNAcβ1-3(GlcNAcβ1-6)GalNAcα1-PAA	Glycotech	01-089
63	Forssman	PAA	GalNAcα1-3GalNAcβ1-PAA	Glycotech	01-026
64	Core6	PAA	GlcNAcβ1-6GalNAcα1-PAA	Glycotech	01-072
65	Core8	PAA	Galα1-3GalNAcα1-PAA	Glycotech	01-028
66	[3'S]Core1	PAA	(3OSO$_3$)Galβ1-3GalNAcα1-PAA	Glycotech	08-069
67	Galβ-Core3	PAA	Galβ1-4GlcNAcβ1-3GalNAcα1-PAA	Glycotech	01-116
68	Asialo-BSM	Glycoprotein	Asialo bovine submaxillary mucin (Tn)	Sigma	M3895 (Acid-
69	Asialo-GP	Glycoprotein	Asialo human glycophorin MN (T)	Sigma	A9791 (Acid-
70	STn	PAA	Neu5Acα2-6GalNAcα1-PAA	Glycotech	01-059
71	STn (Gc)	PAA	Neu5Gcα2-6GalNAcα1-PAA	Glycotech	01-107
72	ST	PAA	Neu5Acα2-3Galβ1-3GalNAcα1-PAA	Glycotech	01-088
73	Siaα2-6Core 1	PAA	Galβ1-3(Neu5Acα2-6)GalNAcα1-PAA	Glycotech	01-113
74	BSM	Glycoprotein	Bovine submaxillary mucin (Sialyl Tn)	Sigma	M3895
75	GP	Glycoprotein	Human glycophorin (Disialyl T and sialyl Tn)	Sigma	G5017
76	αGal	PAA	Galα1-PAA	Glycotech	01-003
77	Galα1-2Gal	PAA	Galα1-2Galβ1-PAA	Glycotech	01-056
78	Galα1-3Gal	PAA	Galα1-3Galβ1-PAA	Glycotech	01-018
79	Galα1-3Lac	PAA	Galα1-3Galβ1-4Glcβ1-PAA	Glycotech	01-075
80	Galα1-3LN	PAA	Galα1-3Galβ1-4GlcNAcβ1-PAA	Glycotech	01-079
81	Galα1-4LN	PAA	Galα1-4Galβ1-4GlcNAcβ1-PAA	Glycotech	01-110
82	Melibiose	PAA	Galα1-6Glcβ1-PAA	Glycotech	01-063
83	αGlc	PAA	Glcα1-PAA	Glycotech	01-001
84	βGlc	PAA	Glcβ1-PAA	Glycotech	01-002
85	Maltose	PAA	Glcα1-4Glcβ1-PAA	Glycotech	01-054
86	HA	BSA	Hyaluronic acid-BSA	Seikagaku	400720
87	CSA	BSA	Chondroitin Sulfate A-BSA	Seikagaku	400655
88	CSB	BSA	Chondroitin Sulfate B-BSA	Seikagaku	400660
89	HS	BSA	Heparan Sulfate-BSA	Seikagaku	400700
90	HP	BSA	Heparin-BSA	Calbiochem	375095
91	KS	BSA	Keratan Sulfate-BSA	Seikagaku	400760
92	αRha	PAA	Rhamnoseα1-PAA	Glycotech	01-008
93	Mannan (SC)	Glycoprotein	*S. cerevisiae* mannan	Sigma	M7504
94	Mannan (CA)	Glycoprotein	*C.albicans* mannan	Takara	MG001
95	Zymosan	Glycoprotein	Zymosan	Sigma	Z4250
96	Chitobiose	PAA	GlcNAcβ1-4GlcNAcβ1-PAA	Glycotech	08-057
97	BSA	BSA	-	Sigma	A7638
98	Negative PAA	PAA	-	Glycotech	01-000
